# Artificial intelligence–powered chatbots for oral anticancer drug patient information: an assessment of quality

**DOI:** 10.1007/s00210-026-05561-w

**Published:** 2026-06-12

**Authors:** Sophie M. Sametinger, Renke Maas, Hagen F. Nicolaus, Wahram Andrikyan

**Affiliations:** 1 Institute of Experimental and Clinical Pharmacology and Toxicology, Friedrich-Alexander-Universität Erlangen-Nürnberg, Fahrstr. 17, 91054 Erlangen, Germany; 2 FAU NeW – Research Center New Bioactive Compounds, Friedrich-Alexander-Universität Erlangen-Nürnberg, Erlangen, Germany; 3 Universitätsklinikum Erlangen, Erlangen, Germany

**Keywords:** Artificial intelligence, Oral anticancer drugs, Drug safety, Large language model, Medication safety, Patient safety

## Abstract

The increasing use of oral anticancer drugs (OADs) in cancer therapy shifts greater responsibility towards patients, thereby also placing a higher informational burden on them. While intensified pharmacological/pharmaceutical care programs have proven beneficial for patients undergoing OAD treatment, their universal availability is currently limited. Given that patients frequently seek health information online, AI-powered chatbots may present a promising resource to address these increasing, yet often unmet information needs. This study aims to evaluate the readability, completeness of relevant information, and accuracy provided by AI-powered chatbots in response to patient questions about OAD treatment. Microsoft Bing’s Copilot and Google’s Gemini were queried in June 2024 on four patient questions regarding ten commonly prescribed and ten recently approved OADs in triplicate. Readability of chatbot answers was assessed using the Flesch reading-ease score (scale 0–100). Completeness of relevant information and accuracy were evaluated based on corresponding standardized written patient information materials. Both chatbots’ answers demonstrated low readability according to the overall mean Flesch reading-ease scores of 38.8 (Copilot) and 50.9 (Gemini). Overall median completeness of relevant information of Copilot’s and Gemini’s answers was 61.1% (IQR, 35.3–78.7%) and 73.8% (IQR, 50.0–100.0%), respectively. Conversely, accuracy of chatbot answers was consistently high, with an overall median accuracy of 100.0% (IQR, 83.3–100.0%) for Copilot and 100.0% (IQR, 98.5–100.0%) for Gemini. AI-powered chatbots provide overall accurate information on OADs. However, their moderate completeness of relevant information and low readability may limit their current practical utility in meeting cancer patients’ information need.

**Supplementary Information:**

The online version contains supplementary material available at https://doi.org/10.1007/s00210-026-05561-w.

## Introduction

Cancer patients are confronted with an overwhelming amount of information regarding their diagnosis and anticancer therapy, which often involves complex therapy schedules. On top of that, the emerging use of oral anticancer drugs (OADs) in treatment regimens introduces novel challenges for cancer patients’ need for information: While treatment with OADs offers many advantages such as higher flexibility and autonomy, patients are also faced with higher responsibility regarding their own drug therapy, including drug intake, storage, and side effect management (Schlichtig et al. 2019).

The randomized AMBORA trial (Dürr et al. 2021) demonstrated the benefits of an intensified clinical pharmacological/pharmaceutical care program for cancer patients undergoing OAD treatment. However, currently, such standardized, intensified clinical pharmacological/pharmaceutical care programs are not universally available to patients receiving OADs (Tibensky et al. 2022).

Research has indicated that cancer patients in general search the web for further cancer information beyond their regular healthcare professional consultation (van Eenbergen et al. 2020). With the rise of artificial intelligence (AI), AI-powered chatbots could help meet patients’ demand for information, particularly in resource-poor settings with limited access to specialized care programs on OADs.

Quality of information in clinical oncology provided by large language models (LLMs), including AI-powered chatbots, has been subject to intensive research in the past (Carl et al. 2024; Chen et al. 2025). However, these previous studies have primarily focused on using LLMs to support healthcare professionals, overlooking its utilization by patients (Carl et al. 2024). Thus, a standardized evaluation on AI-powered chatbots addressing the patient perspective is required, with a particular emphasis on OAD treatment.

The objective of this study is to close this gap by evaluating the readability, completeness of relevant information, and accuracy of chatbot answers given to patient questions regarding OAD treatment using Microsoft Bing’s Copilot and Google’s Gemini, the two leading search engines with integrated AI-powered chatbot features.

## Methods

This study followed the Transparent Reporting of a multivariable model for Individual Prognosis Or Diagnosis (TRIPOD)-LLM reporting guideline for studies using LLMs (Gallifant et al. 2025). The study was conducted in Germany and did not involve research subjects or personalized data as defined by German law or §15 of the Bavarian professional code of physicians (Bayerische Landesärztekammer 2020). Thus, an approval by an ethics board was neither required nor applicable.

### Patient question selection

For simulation of patients consulting chatbots for OAD treatment information, we identified and selected four key patient questions based on standardized OAD fact sheets used in the AMBORA competence and consultation center in routine clinical care, publicly available for healthcare professionals (Cuba et al. 2023; Universitätsklinikum Erlangen 2026). These fact sheets contain information comprehensible for laypersons for each OAD and have been developed in accordance with published guidelines and recommendation (Dürr et al. 2021). The four selected questions covered instructions for precautions, proper drug handling, side effects, and drug/food interactions (Table 1). These questions are referred to as “patient questions” in the following.

**Table 1:** Patient questions and oral anticancer drug selection

Questions		
1. Are there any precautions to take when taking [drug]?		
2. How do I store [drug]?		
3. What are the side effects of [drug] and when should I consult a doctor?		
4. Are there any drug or food interactions with [drug]?		
Oral anticancer drugs (OADs)		
Pharmacological class	Most recently approved drugs	Most commonly prescribed drugs
Hormone receptor antagonist	Elacestrant	Tamoxifen
Alkylating agent	Azacitidine	Temozolomide
Antimetabolite	Decitabin/cedazuridine	Capecitabine
Immunomodulatory drug	Pomalidomide	Lenalidomide
CDK 4/6 inhibitor	Abemaciclib	Palbociclib
PARP inhibitor	Talazoparib	Olaparib
VEGFR inhibitor	Tivozanib	Nintedanib
EGFR inhibitor	Dacomitinib	Osimertinib
MEK inhibitors	Selumetinib	Trametinib
BCR-ABL inhibitor	Asciminib	Imatinib

*OAD*, oral anticancer drug; *CDK4/6*, cyclin dependent kinase 4/6; *PARP*, poly (ADP-ribose) polymerase; *VEGFR*, vascular endothelial growth factor receptor; *EGFR*, epidermal growth factor; *MEK*, mitogen-activated protein kinase; *BCR-ABL*, fusion protein t(9;22).

### Drug selection

We selected 20 drugs in total by first defining ten pharmacological classes and then choosing the most frequently prescribed as well as the most recently approved drugs for each class. This approach ensured a balanced representation across pharmacological classes and prevented the analysis from being skewed towards only a few drug groups. The top ten most commonly prescribed OADs were selected based on the pharmaceutical prescriptions report 2023 of statutory health insurances in Germany analyzing prescriptions of over 70 million insured people (Ludwig et al. 2023). The ten most recently approved OADs referred to the drug approvals by the European Medicines Agency (EMA) (2026) until May 2024.

### Prompt settings

The present study was conducted using Microsoft Bing’s Copilot (based on Prometheus AI and GPT-4) (Microsoft 2023) and Google’s Gemini (Gemini 1.5 Flash) (Gemini API 2024). Both chatbots were used in their respective browser application without further adjustment of preset settings. To ensure standardized prompts and generalizability of chatbot answers, a virtual private network (VPN) was used with the location set to New York City, USA (Windscribe 2026), and all prompts (i.e., queries) were entered in English language into a new chat conversation. To account for the variability of generated chatbot answers, each prompt was queried in triplicate. Both chatbots were queried on the four patient questions regarding all 20 OADs included in the study. Consequently, a total of 480 answers were generated and subsequently analyzed. All chatbot answers were generated in June 2024.

### Assessment of references

To receive an impression of the reliability of the sources, references provided by chatbots when answering patient questions were documented. Microsoft Bing’s Copilot directly references all its sources in its answers (Mehdi 2023). In contrast, at the time of analysis, Google’s Gemini did not provide any sources in its answers. Instead, it allows users to double-check responses by highlighting passages, depending on whether Google Search finds similar content: (1) Green highlights indicate content that is likely similar to the generated passages. A web link is provided, but it is not necessarily the original source that Gemini used to generate its answer. (2) Orange highlights indicate content that is likely to differ from the generated passages, or it did not find relevant content. A link is provided, if available. (3) No highlights indicate that information is insufficient to evaluate the generated passages (Gemini Apps Help 2026). For assessment of Google Gemini’s sources, only references provided with green highlights were used to compensate for the absence of references in the initially generated chatbot answer.

### Assessment of performance metrics

#### Readability

To objectify readability of chatbot answers, the “Flesch reading-ease score” was calculated, which estimates the educational level required to comprehend a given text by a score between 0 and 100. High scores indicate that texts are extremely easy to read, whereas low scores indicate that texts are highly complex necessitating a high educational level for their comprehension (Good Calculators 2026). Conversion tables were used to translate the score into educational levels (Jindal and MacDermid 2017).

#### Number of statements, completeness of relevant information, and accuracy

The newest written information provided by the fact sheets made available by the AMBORA competence and consultation center (Universitätsklinikum Erlangen 2026) served as the evaluation dataset for the assessment of completeness of relevant information and accuracy of chatbot answers. Initially, patient questions were answered utilizing the information given in the fact sheets and these answers were subsequently grouped into statements. Statements were defined as pharmacological or medical propositions regarding a specific topic. The number of statements to answer a patient question using the information provided by the fact sheets was documented.

Accordingly, chatbot answers were grouped into statements. To assess the completeness of relevant information of a chatbot answer, statements were matched with their corresponding statements in the evaluation dataset, irrespective of their correctness.

The assessment of accuracy of chatbot answers was performed following the evaluation framework by Andrikyan et al. (2025). In essence, matching statements were classified into three categories: true (100% accuracy), partially true (50% accuracy), or false statements (0% accuracy). Classification was determined by the degree to which the statement was congruent with the matching statement in the evaluation dataset (Supplemental Table S1). The accuracy of a given chatbot answer resulted from the arithmetic mean of all true, partially true, and false statements within an answer. In addition, this framework was applied for evaluation of green and orange highlights of Gemini’s double-check feature. Initial evaluation of all statements was conducted by a medical student (S.M.S.) with training in pharmacology and subsequently reviewed by a clinical pharmacist (W.A.) and a physician with expertise in pharmacology (H.F.N.).

### Statistical analysis

Microsoft Bing’s Copilot and Google’s Gemini were queried in triplicate for each patient question and OAD. For further assessment, the mean values of the performance metrics for each triplet were used. In instances where a patient question queried in triplicate yielded less than three generated answers from a chatbot due to its refusal to answer, the mean values of the given answers were used. To evaluate readability, completeness of relevant information, and accuracy, the median and interquartile ranges (IQRs) as well as arithmetic mean and sample standard deviation (SD) were calculated. Statistical analysis and graphical visualization were performed using Microsoft Excel (Version 16.103.3, RRID:SCR_016137).

## Results

Both Microsoft Bing’s Copilot and Google’s Gemini were queried about the four patient questions regarding 20 OADs in triplicate. This resulted in a total of 240 chatbot answers with 1525 statements given by Copilot and 231 answers with 1694 statements given by Gemini, the latter refusing to answer 9 patient questions. In detail, Gemini did not provide any answer to a patient question asked in triplicate in one instance. Notably, in two instances, Gemini refused to generate an answer to only one of the three anticipated answers, and in two instances, it refused to generate two of the three anticipated answers.

### References

Copilot cited 50 different websites with a total number of 1141 citations in 240 generated chatbot answers (Supplemental Table S2). After using Gemini’s double-checking feature, the chatbot referenced 120 different websites with a total number of 1080 citations for green highlights in 231 chatbot answers.

### Performance metrics

Assessment of the following performance metrics was based on mean values of each triplet chatbot answer to the four patient questions regarding 20 OADs.

#### Readability

**Fig. 1:**
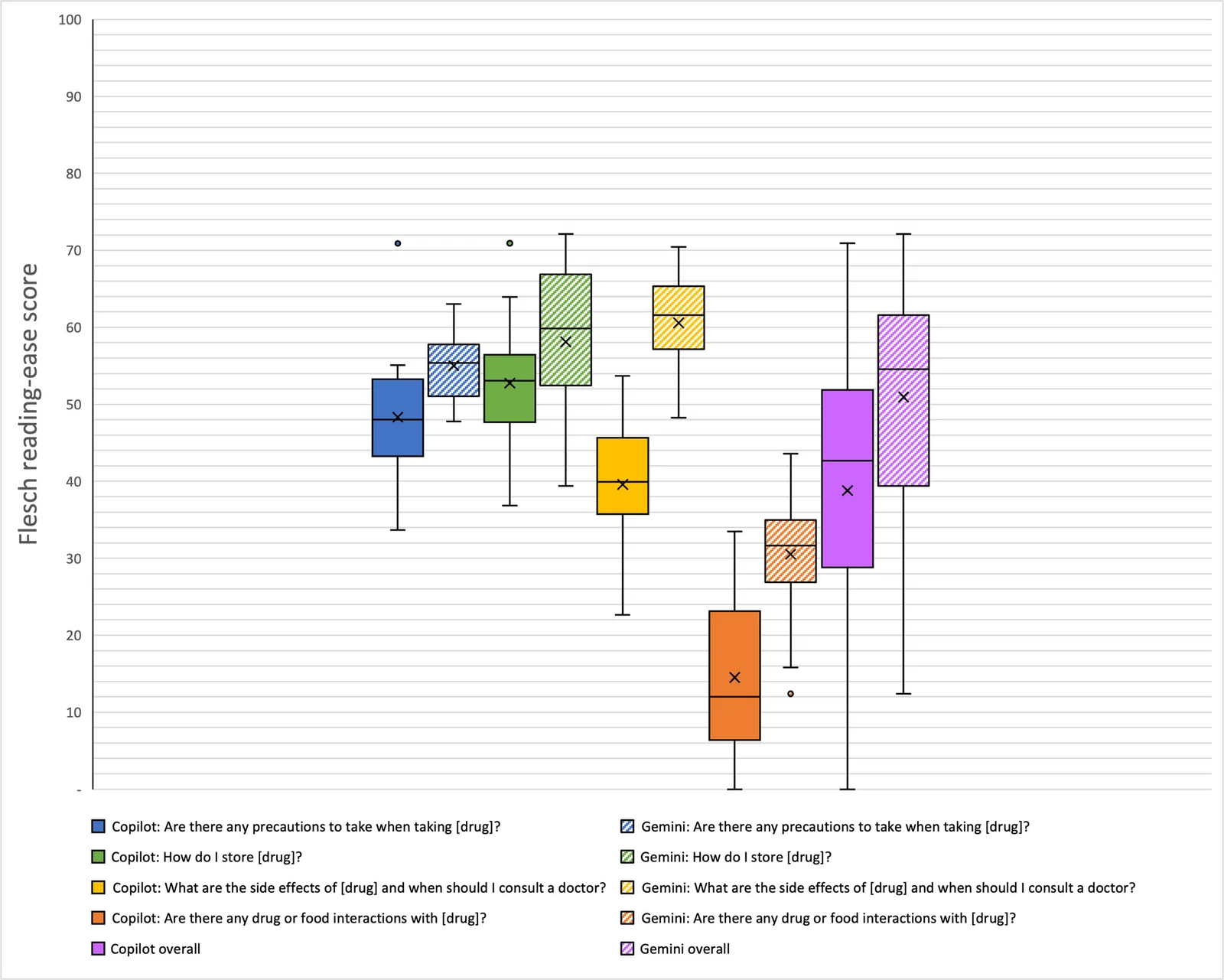
Readability of Copilot and Gemini chatbot answers to four patient questions regarding 20 oral anticancer drugs. Readability of chatbot answers was calculated using the “Flesch reading-ease score.” Box plots show median and interquartile range determined by exclusive median quartile calculation. Whiskers are plotted by using the Tukey method, and the mean is displayed by the “x” symbol. Statistical outliers are shown as dots

Overall and stratified readability of chatbot answers to each patient question is shown in Fig. 1 and Supplemental Table S3.

Overall mean (SD) Flesch reading-ease score of Copilot’s chatbot answers was 38.8 (17.0), indicating an overall high educational level necessitated by the reader (college level). Gemini’s chatbot answers showed an overall mean (SD) Flesch reading-ease score of 50.9 (14.0) is consistent with a text that is fairly difficult to read and requires an educational level of 10th to 12th grade.

#### Number of statements

The overall median number of statements to answer a patient question in the evaluation dataset was 4.0 (IQR, 3.0–8.3) (Supplemental Table S3). In comparison, Copilot’s median number of statements per chatbot answer was 5.0 (IQR, 3.3–7.1), whereas Gemini’s median number of statements was 4.3 (IQR, 4.0–7.3).

#### Completeness of relevant information

**Fig. 2:**
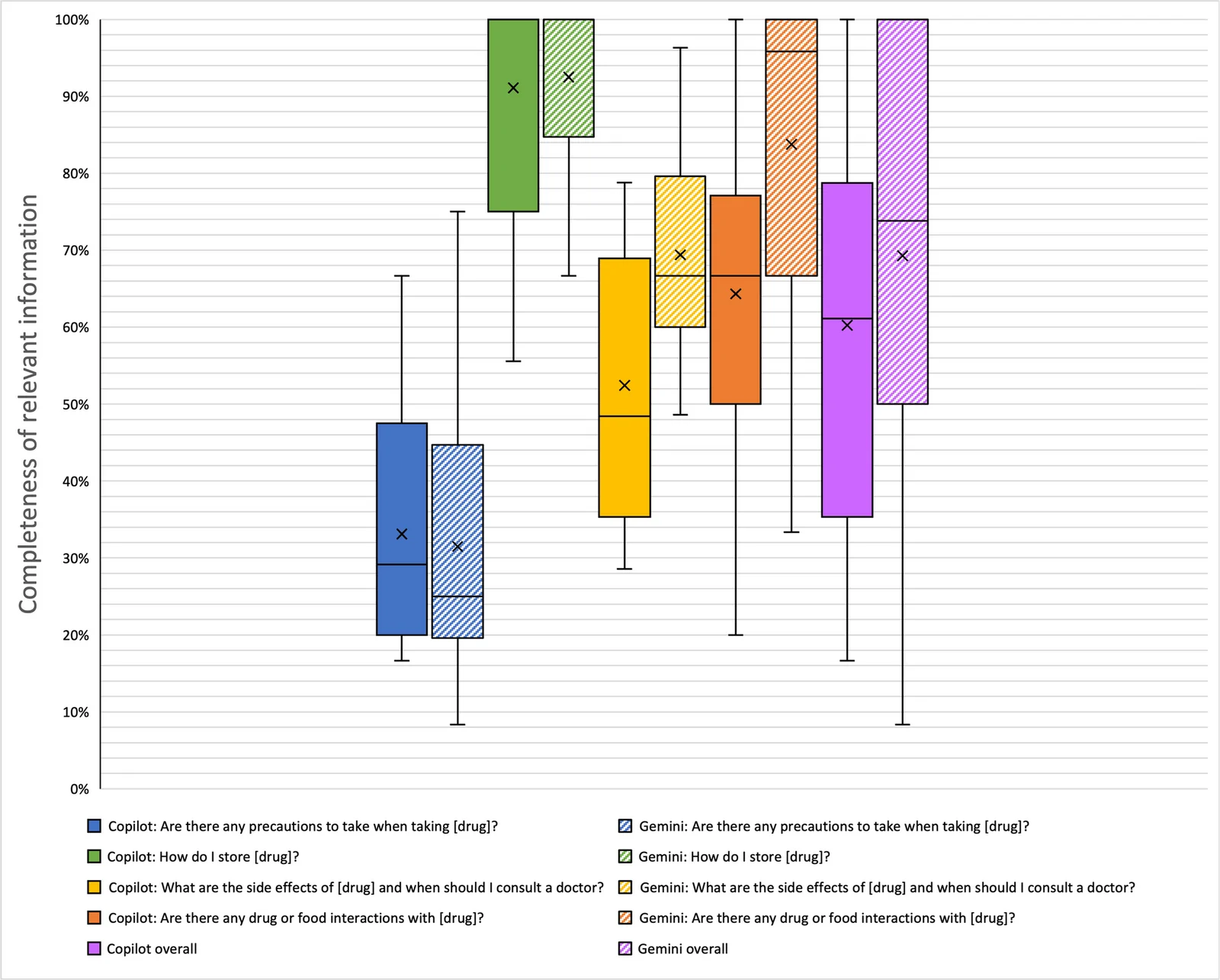
Completeness of relevant information of Copilot and Gemini chatbot answers to four patient questions regarding 20 oral anticancer drugs. Completeness of relevant information was defined as the percentage of matching statements of the chatbot answers in comparison to OAD information of the evaluation dataset retrieved from OAD fact sheets used in the AMBORA competence and consultation center. Box plots show median and interquartile range determined by exclusive median quartile calculation. Whiskers are plotted by using the Tukey method and the mean is displayed by the “x” symbol. Statistical outliers are shown as dots

Overall and stratified completeness of relevant information of chatbot answers to each patient question is shown in Fig. 2 and Supplemental Table S3.

Overall median completeness of relevant information of Copilot’s answers was 61.1% (IQR, 35.3–78.7%) with a mean (SD) of 60.2% (27.4%). Gemini’s answers demonstrated an overall median completeness of relevant information of 73.8% (IQR, 50.0–100.0%) with a mean (SD) of 69.3% (28.4%). Both Copilot’s and Gemini’s chatbot answers showed the highest average completeness of relevant information for patient question 2 (*How do I store [drug]?*), whereas question 1 (*Are there any precautions to take when taking [drug]?*) was answered with the lowest average completeness of relevant information by both chatbots.

#### Accuracy

**Fig. 3:**
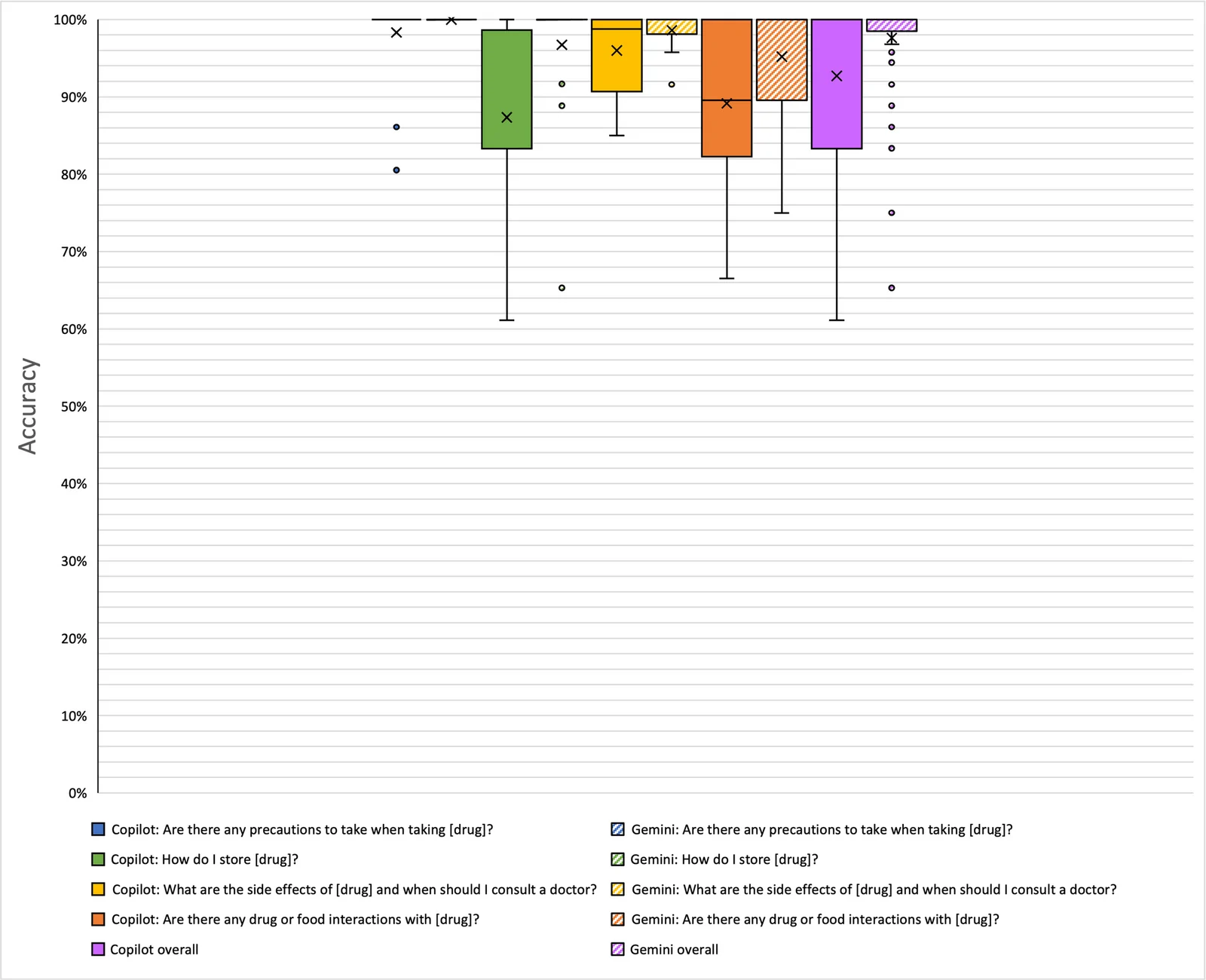
Accuracy of Copilot and Gemini chatbot answers to four patient questions regarding 20 oral anticancer drugs. Accuracy was defined as the weighted sum of true, partially true and false chatbot statements in comparison to matching OAD information of the evaluation dataset retrieved from OAD fact sheets used in the AMBORA competence and consultation center. Box plots show median and interquartile range determined by exclusive median quartile calculation. Whiskers are plotted by using the Tukey method, and the mean is displayed by the “x” symbol. Statistical outliers are shown as dots. Some box plots appear as horizontal lines due to high accuracy scores and low variability of data

Overall and stratified accuracy of chatbot answers to each patient question is shown in Fig. 3 and Supplemental Table S3.

Due to the absence of matching statements with the evaluation dataset, i.e., a completeness of relevant information of 0.0%, the assessment of accuracy was not feasible in one of 240 (0.4%) of Copilot’s answers and in three of 231 (1.3%) of Gemini’s answers.

Evaluation of Copilot’s answers demonstrated an overall median accuracy of 100.0% (IQR, 83.3–100.0%) with a mean (SD) of 92.7% (9.3%). Overall median accuracy of Gemini’s answers was 100.0% (IQR, 98.5–100.0%) with a mean (SD) 97.6% (5.8%). Highest average accuracy was observed for questions 1 (*Are there any precautions to take when taking [drug]?*) in both Copilot’s and Gemini’s chatbot answers. Conversely, Copilot’s lowest average accuracy was assessed for question 2 (*How do I store [drug]?*), while Gemini’s answers to question 4 showed the lowest average accuracy. Notably, not a single of Copilot’s or Gemini’s answers in their entirety showed an accuracy of 0.0%. Moreover, the two chatbots’ answer quality did not differ substantially when comparing their performance on commonly prescribed and recently approved drugs (Supplemental Table S4).

#### Double-checking of chatbot answers—green and orange highlights

Application of Gemini’s double-check feature to all 231 generated chatbot answers resulted in the labeling of 1842 green highlights and 305 orange highlights. Of these, 985 (53.5%) green highlights and 185 (60.6%) orange highlights were matching the evaluation dataset, thereby meeting the criteria for accuracy evaluation (Table 2). For green highlights, 981 (99.6%) were rated as true, only four (0.4%) were rated as false, and none was rated as partially true. Notably, orange highlights showed an overall high accuracy with 185 (100.0%) orange highlights rated as true, and none rated as partially true or false.

**Table 2:** Accuracy of green and orange highlights provided in Gemini’s double-checked answers

Evaluation	Green highlights*	Orange highlights**
True	981	185
Partially true	0	0
False	4	0
Total	985	185

*Green highlights indicate content that is likely similar to the generated passages

**Orange highlights indicate content that is likely to differ from the generated passages, or it did not find relevant content

## Discussion

### Key findings

To the best of our knowledge, this is the first study to evaluate the quality of chatbot answers to patient questions regarding commonly prescribed and recently approved OADs. Both Microsoft Bing’s Copilot and Google’s Gemini generated highly accurate answers, regardless of whether chatbots were queried on the most commonly prescribed or the most recently approved OADs. However, chatbot answers showed an overall moderate completeness of relevant information and low readability. This may be of particular concern for cancer patients, as it could potentially introduce significant risks to their drug safety.

#### Variability of chatbot answers

In this study, patients seeking information on OAD treatment from chatbots were simulated by prompting Copilot and Gemini with easy-worded and clear patient questions based on standardized OAD fact sheets used in the AMBORA competence and consultation center. To account for variability of answers, both chatbots were queried in triplicate. Even though patient questions in triplicate were identical, Gemini occasionally refused to generate one, two, or even all of the three anticipated answers. It is known that chatbots are capable of producing inaccurate yet convincing responses. To address this issue, chatbots can be designed to refuse an answer in cases of uncertainty (Wen et al. 2025). Considering this, by providing a response, the user is led to believe that the generated information holds a certain degree of accuracy. Consequently, an inconsistency in the generation or refusal to respond, despite being queried on the identical question, may mislead the user and could pose a safety risk.

#### References

Overall, Copilot referenced predominantly reliable, peer reviewed, and evidence-based sources for the generation of its answers, as the majority of sources cited consist of drugs.com (Drugs.com n.d.) and mayoclinic.org (Mayo Foundation for Medical Education and Research n.d.), together accounting for 40.2% in total. Gemini did not disclose any sources for its generated answers during the study period. Instead, Gemini allows to double-check its answers by searching for similar content on the internet and highlighting passages in its answer accordingly. The green highlights, indicating similar content, were provided with webpages that were considered mainly reliable. Orange highlights, however, indicate potentially conflicting information on the internet. Interestingly, analysis of highlights matching with the evaluation dataset revealed high accuracies for both green and orange highlights. Gemini’s double-check feature may help encourage users to verify generated chatbot answers. However, the color-coding system of highlights might not serve as an effective indicator for accuracy, as the coloring also varies based on the accuracy of the webpage content.

#### Readability

In our analysis, Copilot and Gemini demonstrated an overall low readability, with mean Flesch reading-ease scores of 38.8 and 50.9, respectively. These scores translate to a college educational level (Copilot) and an educational level of 10th to 12th grade (Gemini) necessitated by the reader to comprehend the generated answers.

Calculation of the Flesch reading-ease score is based on the number of syllables, words, and sentences (Good Calculators 2026). The American Medical Association (AMA) recommends health information, such as patient information leaflets, to use simple language with one- or two-syllable words, short paragraphs, and sentences, congruent with a 6th grade educational level in the USA (Weiss 2006). Answers of both chatbots did not meet these proposed standards for health information. The low readability of chatbot answers could limit the potential benefits of using chatbots as a source for cancer information, given that low health literacy is linked to higher all-cause mortality among cancer patients (Al Hussein Al Awamlh et al. 2025). Nevertheless, it can be assumed that cancer patients acquire an above-average health literacy in the course of their condition as a result of their substantial exposure to information regarding their diagnosis and therapy. In this context, patients may find benefit from answers generated by AI-powered chatbots.

#### Completeness of relevant information and accuracy

Despite the slightly higher median number of statements per answer to a patient question employed by both Copilot and Gemini compared to the evaluation dataset, the completeness of relevant information of answers of both chatbots was only moderate, with an overall median completeness of relevant information of 61.1% and 73.8%, respectively. This result indicates the challenge of providing exhaustive information on OADs in a summarized form. As excessive and clinically irrelevant drug information can negatively affect patient adherence and safety (Herber et al. 2014), further research is needed to clarify the impact of AI-generated drug information. Both chatbots performed similarly in terms of individual questions. The lowest completeness of relevant information was observed for patient question 1 regarding precautions to take when taking an OAD. This finding aligns with prior research that indicated chatbots frequently struggle to provide complete answers to open-ended queries (Andrikyan et al. 2025).

Conversely, the overall median accuracy of chatbot answers was consistently high. It is noteworthy that both chatbots demonstrated the highest accuracy in their answers to the patient question 1, which had the lowest completeness of relevant information. This suggests that if statements matched with the evaluation dataset, they were predominantly accurate. Furthermore, the comparative analysis of both Copilot’s and Gemini’s answers revealed negligible differences in accuracy when evaluating commonly prescribed and recently approved OADs (Supplemental Table S4). This was of particular interest, because it is expected that there is more experience and, thus, more information on dealing with commonly prescribed OADs compared to recently approved drugs. On the other hand, recent drug approvals demand more extensive studies due to higher regulatory requirements, leading to more information on recently approved drugs (U.S. Food & Drug Administration (FDA) 2025) at time of their approval. Additionally, information on older drugs retrieved from the internet poses a risk to be outdated (Andrikyan et al. 2025). This study shows that AI-powered chatbots are able to generate highly accurate answers, even in the early phases following drug approval for the respective OAD.

In rare instances, differences between the U.S. Food and Drug Administration (FDA) and EMA drug labels may have led to statements which did not align with the evaluation dataset, resulting in low accuracy. For example, at the time of chatbot answer generation, the EMA drug label for trametinib indicated that tablets should be stored at room temperature, whereas the FDA drug label mandated the storage at 2 to 8 °C (U.S. Food & Drug Administration (FDA) 2024; European Medicines Agency (EMA) 2024). When the chatbot was queried on the appropriate drug storage (patient question 2), it recommended to store trametinib refrigerated adhering to the FDA drug label. To avoid such potential consequences due to the discrepancies of different information sources, chatbots should clearly demonstrate their sources of answer generation.

#### Question selection and reference answer

Concerning the question about side effects, we relied on the standardized OAD fact sheets as reference, as they emphasize on the most clinically relevant and comprehensible ones. In contrast to patient information leaflets, which may contain extensive or even partially misleading side-effect listings (Barker and Faasse 2023), the standardized OAD fact sheets were assumed to include primarily actual and clinically relevant side effects. Consequently, conventional patient information leaflets are only of limited suitability as reference for assessing the quality of relevant information of chatbot answers and were therefore not analyzed in this study.

Furthermore, the standardized OAD fact sheets primarily list over-the-counter (OTC) drug products regarding drug interaction management, which patients often use without telling their physicians and hence represent a clinically important aspect for medical consultations (Tsele-Tebakang 2022). Our broader question wording may have influenced the evaluation of completeness of relevant information regarding drug interaction management.

### Unmatched statements

Often, Copilot’s and Gemini’s answers were extensive and elaborate. Because they frequently did not correspond to any statement in the reference answer, they consisted partly of clinically irrelevant information and therefore fell into the category of “unmatched statements” as defined in the present study. This tendency might partly be explained by the fact that LLMs are able to draw on patient information leaflets, which often contain extensive information that is not always supported by compelling evidence and can therefore have a negative impact on compliance, compromising patient’s safety (Reith et al. 2026). The present findings emphasize the importance of the clinical relevance of the evaluation dataset when assessing AI-generated medical answers in clinical oncology. The methodology used in the present work may provide a useful blueprint for future assessments in this field, as the rapid progress in AI warrants continuous monitoring.

## Limitations

Our study had several limitations. No genuine patient engagement was assessed in this study. Instead, patients consulting chatbots for OAD treatment information were simulated by prompting chatbots with patient questions that were retrieved from the content of standardized OAD fact sheets. These questions were queried in triplicate, yet they were neither permutated nor designed as an authentic conversation, in which a patient could engage with in a real-world setting. Thus, the observed spectrum of chatbot answers was less varied than in a real-world setting. Furthermore, the actual patient experience including the perceived helpfulness of chatbot answers was not evaluated. Additionally, the risk of patient harm if patients follow the chatbot’s given recommendations was not directly assessed. However, a potential patient harm can be implied due to the immediate consequences of incomplete or inaccurate advice on OAD management. Lastly, querying the chatbots each question in triplicate helps account for variability in their answers. Yet, a thorough assessment of variability and its possible consequences for patients was beyond the scope of the present study and not among the objectives. Nevertheless, future investigations would be both valuable and important, as variability in chatbot answers might raise concerns regarding patient safety.

## Outlook

Comprehensive reviews (Carl et al. 2024; Chen et al. 2025) on AI-powered chatbots applied in clinical oncology settings have demonstrated performance variances depending on, among other factors, the examined oncological subfield and version of the chatbot. While previous studies have primarily focused on using AI-powered chatbots to support healthcare professionals, our study addressed the patient perspective with an emphasis on OAD treatment. We observed an overall high accuracy but only moderate completeness of relevant information of chatbot answers regarding patient questions on OAD management. Copilot’s and Gemini’s conversational capabilities enable a vast variety of applications, potentially tempting patients to also search for information on their medication from these chatbots. However, it should be noted that neither Copilot nor Gemini was intended to be implemented directly into clinical care and may pose a risk for medication safety, as they retrieve information from the whole internet.

To address these risks, specific models designed for use in clinical care should be trained in alignment with regulatory standards (Federal Register 2024). Such a model should be based on reliable sources for output generation, including databases that are continuously updated and reviewed.

## Conclusion

The present study shows that modern AI-powered chatbots can generate overall accurate answers to patient questions on OAD treatment information. However, the moderate completeness of relevant information and overall low readability of chatbot answers demonstrate the challenge of providing comprehensive yet concise information on OADs suitable for laypersons. Thus, the ability of AI-powered chatbots in addressing cancer patients’ high demand for information on OADs may currently be limited in practice. However, the capabilities of AI models are rapidly evolving, which underlines the need for regular benchmarking of the performance of chatbots applied in various clinical oncology settings. In this respect, this study may serve as a blueprint for future (re-)evaluations of AI-generated drug information for patients.

## Supplementary Information

Below is the link to the electronic supplementary material.

ESM 1 (DOCX 39.9 KB)

## Data Availability

The data that support the findings of this study are available from the corresponding author upon reasonable request.
