# Supplementary material for: Artificial intelligence–powered chatbots for oral anticancer drug patient information: an assessment of quality
Source: Naunyn Schmiedebergs Arch Pharmacol. 2026 Jun 12;399(12):18779–89. doi: 10.1007/s00210-026-05561-w (PMC13593639; doi:10.1007/s00210-026-05561-w)
Supplement: Supplementary file 1 [file 210_2026_5561_MOESM1_ESM.docx]

**SUPPLEMENTARY MATERIAL**

**Naunyn-Schmiedeberg's Archives of Pharmacology**

**Artificial intelligence-powered chatbots for oral anticancer drug patient information: An assessment of quality**

Sophie M. Sametinger, Renke Maas, Hagen F. Nicolaus, Wahram Andrikyan

Address for correspondence: Wahram Andrikyan, Institute of Experimental and Clinical Pharmacology and Toxicology, Friedrich-Alexander-Universität Erlangen-Nürnberg, Fahrstr. 17, 91054 Erlangen, Germany. E-mail: wahram.andrikyan@fau.de

**Supplemental Table S1. Example assessments for true, partially true, and false Copilot and Gemini chatbot statements.**

| **Patient question** | **Chatbot statement** | **Reference database statement** | **Evaluation** |
| --- | --- | --- | --- |
| Are there any precautions to take when taking decitabin/cedazuridin | Swallow Whole: Swallow the tablet whole; do not cut, crush, or chew it. | Do not suck, crush, chew, or cut the tablets.  Lutschen, zerdrücken, zerkauen, zerschneiden oder zerbrechen Sie Ihre Tabletten nicht. | **true** |
| Are there any precautions to take when taking capecitabin? | Follow procedures for proper handling and disposal of hazardous drugs. | Wash your hands before and after the intake.  Relatives should wear disposable gloves when handling the cancer medication.  Waschen Sie sich vor und nach der Einnahme die Hände. Angehörige sollten beim Umgang mit dem Krebsmedikament Einmalhandschuhe tragen | **partially true** |
| How do I store trametinib? | Trametinib should be stored as follows:  Tablets: Keep them in the refrigerator (but do not freeze) | Store at room temperature, protected from sunlight (not on the windowsill), moisture (not in the bathroom), and heat (not in the kitchen).  Bei Raumtemperatur, vor Sonneneinstrahlung (nicht auf der Fensterbank), Feuchtigkeit (nicht im Bad) und Hitze (nicht in der Küche) geschützt lagern. | **false** |

**Supplemental Table S2. All references cited by Copilot and Gemini chatbot.**

| **Copilot** |  | **Gemini** |  |
| --- | --- | --- | --- |
| **No. of citations** | **Website** | **No. of citations** | **Website** |
| **1141 (100%)** | **total** | **1080 (100%)** | **total** |
| 255 (22.3%) | drugs.com | 195 (18.1%) | mayoclinic.org |
| 204 (17.9%) | mayoclinic.org | 70 (6.5%) | [cancerresearchuk.org](http://www.cancerresearchuk.org/) |
| 109 (9.6%) | wikipedia.org | 63 (5.8%) | [webmd.com](http://www.webmd.com/) |
| 82 (7.2%) | webmd.com | 58 (5.4%) | medlineplus.gov |
| 80 (7.0%) | mskcc.org | 56 (5.2%) | [drugs.com](http://www.drugs.com/) |
| 58 (5.1%) | msn.com | 47 (4.4%) | my.clevelandclinic.org |
| 54 (4.7%) | medlineplus.gov | 44 (4.1%) | [cancer.gov](http://www.cancer.gov/) |
| 25 (2.2%) | rxlist.com | 44 (4.1%) | macmillan.org.uk |
| 24 (2.1%) | everydayhealth.com | 43 (4.0%) | pubmed.ncbi.nlm.nih.gov |
| 23 (2.0%) | my.clevelandclinic.org | 33 (3.1%) | [oncolink.org](http://www.oncolink.org/) |
| 21 (1.8%) | www.oncolink.org | 22 (2.0%) | [mskcc.org](http://www.mskcc.org/) |
| 20 (1.6%) | www.medicine.com | 20 (1.9%) | [rxlist.com](http://www.rxlist.com/) |
| 18 (1.6%) | www.oralchemoedsheets.com | 18 (1.7%) | reference.medscape.com |
| 12 (1.1%) | go.drugbank.com | 16 (1.5%) | [medicalnewstoday.com](http://www.medicalnewstoday.com/) |
| 11 (1.0%) | navigatingcare.com | 15 (1.4%) | [fda.gov](http://www.fda.gov/) |
| 9 (0.8%) | link.springer.com | 14 (1.3%) | breastcancernow.org |
| 9 (0.8%) | medicinenet.com | 14 (1.3%) | [oralchemoedsheets.com](http://www.oralchemoedsheets.com/) |
| 9 (0.8%) | singhealth.com.sg | 13 (1.2%) | [breastcancer.org](http://www.breastcancer.org/) |
| 7 (0.6%) | bccancer.bc.ca | 13 (1.2%) | [ncbi.nlm.nih.gov](http://www.ncbi.nlm.nih.gov/) |
| 7 (0.6%) | [bing.com](https://bing.com/) | 10 (0.9%) | cstep.cancer.gov |
| 6 (0.5%) | hillman.upmc.com | 10 (0.9%) | go.drugbank.com |
| 6 (0.5%) | accessdata.fda.gov | 9 (0.8%) | [cancercareontario.ca](http://www.cancercareontario.ca/) |
| 6 (0.5%) | goodrx.com | 9 (0.8%) | [gmp-compliance.org](http://www.gmp-compliance.org/) |
| 6 (0.5%) | ons.org | 8 (0.7%) | [tagrisso.com](http://www.tagrisso.com/) |
| 6 (0.5%) | rxwiki.com | 8 (0.7%) | [lynparza.com](http://www.lynparza.com/) |
| 4 (0.4%) | icliniq.com | 7 (0.6%) | [pfizermedicalinformation.com](http://www.pfizermedicalinformation.com/) |
| 3 (0.3%) | dam.upmc.com | 7 (0.6%) | karger.com |
| 3 (0.3%) | doctors.valleyhealth.com | 6 (0.6%) | [healthline.com](http://www.healthline.com/) |
| 3 (0.3%) | foodforbreastcancer.com | 6 (0.6%) | academic.oup.com |
| 3 (0.3%) | koselugohcp.com | 6 (0.6%) | ascopubs.org |
| 3 (0.3%) | media.revlimid.com | 6 (0.6%) | [inqovi.com](http://www.inqovi.com/) |
| 3 (0.3%) | publications.ashp.org | 6 (0.6%) | [ofev.com](http://www.ofev.com/) |
| 3 (0.3%) | reference.medscape.com | 6 (0.6%) | [vizimpro.com](http://www.vizimpro.com/) |
| 3 (0.3%) | rheumatology.org.au | 6 (0.6%) | [wellrx.com](http://www.wellrx.com/) |
| 3 (0.3%) | breastcancer.org | 5 (0.5%) | [kaiserpermanente.org](http://www.kaiserpermanente.org/) |
| 3 (0.3%) | cancer.gov | 5 (0.5%) | [atsjournals.org](http://www.atsjournals.org/) |
| 3 (0.3%) | cancercareontario.ca | 5 (0.5%) | [researchgate.net](http://www.researchgate.net/) |
| 3 (0.3%) | cellsignal.com | 4 (0.4%) | [eviq.org.au](http://www.eviq.org.au/) |
| 3 (0.3%) | ema.europa.eu | 4 (0.4%) | account.allinahealth.org |
| 3 (0.3%) | healthline.com | 4 (0.4%) | wms.org |
| 3 (0.3%) | jax.org | 4 (0.4%) | [medicinesinpregnancy.org](http://www.medicinesinpregnancy.org/) |
| 3 (0.3%) | kidneycancer.org | 4 (0.4%) | [pomalyst.com](http://www.pomalyst.com/) |
| 3 (0.3%) | mdpi.com | 3 (0.3%) | labeling.pfizer.com |
| 3 (0.3%) | med.upenn.edu | 3 (0.3%) | aacrjournals.com |
| 3 (0.3%) | northwell.edu | 3 (0.3%) | medilib.ir |
| 3 (0.3%) | pfizermedicalinformation.com | 3 (0.3%) | oncologypro.esmo.org |
| 3 (0.3%) | practo.com | 3 (0.3%) | [andygaya.com](http://www.andygaya.com/) |
| 3 (0.3%) | safemedication.com | 3 (0.3%) | [bidmc.org](http://www.bidmc.org/) |
| 3 (0.3%) | swagcanceralliance.nhs.uk | 3 (0.3%) | [facingourrisk.org](http://www.facingourrisk.org/) |
| 1 (0.1%) | medicinenet.com | 3 (0.3%) | [healthlinkbc.ca](http://www.healthlinkbc.ca/) |
|  | | 3 (0.3%) | [limamemorial.org](http://www.limamemorial.org/) |
|  |  | 3 (0.3%) | [medical.lilly.com](http://www.medical.lilly.com/) |
|  |  | 3 (0.3%) | [medicineinpregnancy.org](http://www.medicineinpregnancy.org/) |
|  |  | 3 (0.3%) | [medicinenet.com](http://www.medicinenet.com/) |
|  |  | 3 (0.3%) | [ncbi.nlm.nih](http://www.ncbi.nlm.nih/) |
|  |  | 3 (0.3%) | [pharmacytimes.com](http://www.pharmacytimes.com/) |
|  |  | 3 (0.3%) | [scrippslabs.com](http://www.scrippslabs.com/) |
|  |  | 3 (0.3%) | [stjude.org](http://www.stjude.org/) |
|  |  | 3 (0.3%) | [tandfonline.com](http://www.tandfonline.com/) |
|  |  | 3 (0.3%) | [uspharmacist.com](http://www.uspharmacist.com/) |
|  |  | 3 (0.3%) | [verywellhealth.com](http://www.verywellhealth.com/) |
|  |  | 3 (0.3%) | [vinmec.com](http://www.vinmec.com/) |
|  |  | 2 (0.2%) | ibrance.pfizerpro.com |
|  |  | 2 (0.2%) | [gov.uk](http://www.gov.uk/) |
|  |  | 2 (0.2%) | [healthhub.sg](http://www.healthhub.sg/) |
|  |  | 2 (0.2%) | [thebraintumorcharity.org](http://www.thebraintumorcharity.org/) |
|  |  | 2 (0.2%) | bmjopenrespres.bmj.com |
|  |  | 2 (0.2%) | chemocare.com |
|  |  | 2 (0.2%) | chicagocolorectal.com |
|  |  | 2 (0.2%) | dailymed.nlm.nih.gov |
|  |  | 2 (0.2%) | [ftp.uspbpep.com](ftp://ftp.uspbpep.com/) |
|  |  | 2 (0.2%) | medsafe.govt.nz |
|  |  | 2 (0.2%) | mhvidp-prod.myhealth.va.gov |
|  |  | 2 (0.2%) | newsnetwork.mayoclinic.org |
|  |  | 2 (0.2%) | thekingsleyclinic.com |
|  |  | 2 (0.2%) | velindre.nhs.wales |
|  |  | 2 (0.2%) | [boehringer-ingelheim.com](http://www.boehringer-ingelheim.com/) |
|  |  | 2 (0.2%) | [komen.org](http://www.komen.org/) |
|  |  | 2 (0.2%) | [nature.com](http://www.nature.com/) |
|  |  | 2 (0.2%) | [pca.sate.mn.us](http://www.pca.sate.mn.us/) |
|  |  | 2 (0.2%) | [rosswellpark.org](http://www.rosswellpark.org/) |
|  |  | 1 (0.1%) | ashpublications.org |
|  |  | 1 (0.1%) | kunskapsbanken.cancercentrum.se |
|  |  | 1 (0.1%) | [childrensmn.org](http://www.childrensmn.org/) |
|  |  | 1 (0.1%) | bmcpulmmed.biomedcentral.com |
|  |  | 1 (0.1%) | braintumorcenter.ucsf.edu |
|  |  | 1 (0.1%) | en.wikipedia.org |
|  |  | 1 (0.1%) | globalrph.com |
|  |  | 1 (0.1%) | grandaveurgentcare.com |
|  |  | 1 (0.1%) | koselugo.com |
|  |  | 1 (0.1%) | linkspringer.com |
|  |  | 1 (0.1%) | lungfoundation.com.au |
|  |  | 1 (0.1%) | medbroadcast.com |
|  |  | 1 (0.1%) | owise.us |
|  |  | 1 (0.1%) | phoc.navigatingcare.com |
|  |  | 1 (0.1%) | ufhealth.org |
|  |  | 1 (0.1%) | verzenio.lilly.com |
|  |  | 1 (0.1%) | voice.ons.org |
|  |  | 1 (0.1%) | [amoena.com](http://www.amoena.com/) |
|  |  | 1 (0.1%) | [bloodresearch.or.kr](http://www.bloodresearch.or.kr/) |
|  |  | 1 (0.1%) | [crossroadspsychiatric.com](http://www.crossroadspsychiatric.com/) |
|  |  | 1 (0.1%) | [everydayhealth.com](http://www.everydayhealth.com/) |
|  |  | 1 (0.1%) | [gistsupport.org](http://www.gistsupport.org/) |
|  |  | 1 (0.1%) | [health.com](http://www.health.com/) |
|  |  | 1 (0.1%) | [ibrance.com](http://www.ibrance.com/) |
|  |  | 1 (0.1%) | [leedsth.nhs.uk](http://www.leedsth.nhs.uk/) |
|  |  | 1 (0.1%) | [lynparzahcp.com](http://www.lynparzahcp.com/) |
|  |  | 1 (0.1%) | [mdpi.com](http://www.mdpi.com/) |
|  |  | 1 (0.1%) | [mountsinai.org](http://www.mountsinai.org/) |
|  |  | 1 (0.1%) | [myactivehealth.com](http://www.myactivehealth.com/) |
|  |  | 1 (0.1%) | navigatingcare.com |
|  |  | 1 (0.1%) | [nhs.uk](http://www.nhs.uk/) |
|  |  | 1 (0.1%) | [oncnursingnews.com](http://www.oncnursingnews.com/) |
|  |  | 1 (0.1%) | [orserdu.com](http://www.orserdu.com/) |
|  |  | 1 (0.1%) | [psychiatrist.com](http://www.psychiatrist.com/) |
|  |  | 1 (0.1%) | [redcrossblood.org](http://www.redcrossblood.org/) |
|  |  | 1 (0.1%) | [singlecare.com](http://www.singlecare.com/) |
|  |  | 1 (0.1%) | [spandido-publications.com](http://www.spandido-publications.com/) |
|  |  | 1 (0.1%) | [ucsfhealth.org](http://www.ucsfhealth.org/) |
|  |  | 1 (0.1%) | [wheatfieldpediatrics.com](http://www.wheatfieldpediatrics.com/) |

**Supplemental Table S3. Completeness, accuracy, and readability scores for Copilot and Gemini chatbot answers**.

|  | | **No. Statements reference** | **No. Statements Copilot** | **No. Statements Gemini** | **Completeness Copilot [%]** | **Completeness Gemini [%]** | **Accuracy Copilot [%]** | **Accuracy Gemini [%]** | **Readability Copilot** | **Readability Gemini** |
| --- | --- | --- | --- | --- | --- | --- | --- | --- | --- | --- |
| **Overall** | Median | 4.0 | 5.0 | 4.3 | 61.1 | 73.8 | 100.0 | 100.0 | 42.7 | 54.6 |
|  | IQR | 3.0-8.3 | 3.3-7.1 | 4.0-7.3 | 35.3-78.7 | 50.0-100.0 | 83.3-100.0 | 98.5-100.0 | 28.8-51.9 | 39.4-61.6 |
|  | Mean | 7.2 | 6.4 | 7.4 | 60.2 | 69.3 | 92.7 | 97.6 | 38.8 | 50.9 |
|  | SD | 6.5 | 4.5 | 6.0 | 27.4 | 28.4 | 9.3 | 5.8 | 17.0 | 14.0 |
| **Are there any precautions to take when taking [drug]?** | Median | 4.5 | 3.0 | 4.2 | 29.2 | 25.0 | 100.0 | 100.0 | 48.0 | 55.4 |
|  | IQR | 4.0-5.0 | 2.0-4.8 | 3.3-5.3 | 20.0-47.5 | 19.6-44.7 | 100.0-100.0 | 100.0-100.0 | 43.2-53.3 | 51.0-57.8 |
|  | Mean | 4.7 | 3.4 | 4.3 | 33.1 | 31.5 | 98.3 | 100.0 | 48.3 | 55.0 |
|  | SD | 0.9 | 1.4 | 1.5 | 14.5 | 17.9 | 5.2 | 0.0 | 8.0 | 4.2 |
| **How do I store [drug]?** | Median | 3.0 | 4.0 | 4.3 | 100.0 | 100.0 | 83.3 | 100.0 | 53.1 | 59.8 |
|  | IQR | 3.0-3.0 | 3.4-4.0 | 4.0-5.0 | 75.0-100.0 | 84.7-100.0 | 83.3-98.6 | 100.0-100.0 | 47.7-56.5 | 52.4-66.9 |
|  | Mean | 3.1 | 3.8 | 4.4 | 91.1 | 92.5 | 87.3 | 96.7 | 52.7 | 58.1 |
|  | SD | 0.2 | 0.7 | 0.7 | 16.0 | 11.7 | 10.0 | 8.3 | 7.6 | 9.5 |
| **What are the side effects of [drug] and when should I consult a doctor?** | Median | 18.0 | 13.0 | 17.5 | 48.4 | 66.7 | 98.8 | 100.0 | 39.9 | 61.6 |
|  | IQR | 14.0-21.0 | 10.8-15.2 | 16.3-19.7 | 35.3-68.9 | 60.0-79.6 | 90.7-100.0 | 98.1-100.0 | 35.7-45.7 | 57.1-65.3 |
|  | Mean | 17.5 | 13.2 | 17.6 | 52.4 | 69.4 | 96.0 | 98.6 | 39.6 | 60.6 |
|  | SD | 4.8 | 3.6 | 2.4 | 17.0 | 12.4 | 5.1 | 2.1 | 7.4 | 6.2 |
| **Are there any drug or food interactions with [drug]?** | Median | 4.0 | 5.0 | 3.8 | 66.7 | 95.8 | 89.6 | 100.0 | 12.0 | 31.7 |
|  | IQR | 2.3-4.0 | 4.0-6.0 | 3.1-4.0 | 50.0-77.1 | 66.7-100.0 | 82.3-100.0 | 89.6-100.0 | 6.4-23.1 | 26.9-35.0 |
|  | Mean | 3.5 | 5.1 | 3.7 | 64.3 | 83.8 | 89.2 | 95.2 | 14.5 | 30.5 |
|  | SD | 1.1 | 1.3 | 0.7 | 22.5 | 20.3 | 10.9 | 7.3 | 10.4 | 7.5 |

**Supplemental Table S4. Copilot (a) and Gemini (b) chatbot answers performance on commonly prescribed and recently approved OADs.**

**a)**

|  |  | **Copilot** | | | |
| --- | --- | --- | --- | --- | --- |
|  |  | **Completeness commonly prescribed OADs [%]** | **Completeness recently approved OADs [%]** | **Accuracy commonly prescribed OADs [%]** | **Accuracy recently approved OADs [%]** |
| **Overall** | Median | 58.3 | 61.1 | 96.3 | 100.0 |
|  | IQR | 38.8-78.4 | 33.7-82.2 | 83.3-100.0 | 85.3-100.0 |
|  | Mean | 60.4 | 60.1 | 91.7 | 93.7 |
|  | SD | 27.6 | 27.5 | 10.3 | 8.2 |

**b)**

|  |  | **Gemini** | | | |
| --- | --- | --- | --- | --- | --- |
|  |  | **Completeness commonly prescribed OADs [%]** | **Completeness recently approved OADs [%]** | **Accuracy commonly prescribed OADs [%]** | **Accuracy recently approved OADs [%]** |
| **Overall** | Median | 70.4 | 73.8 | 100.0 | 100.0 |
|  | IQR | 49.0-100.0 | 55.0-100.0 | 96.2-100.0 | 100.0-100.0 |
|  | Mean | 68.0 | 70.6 | 96.3 | 98.9 |
|  | SD | 28.9 | 28.1 | 7.5 | 3.0 |
